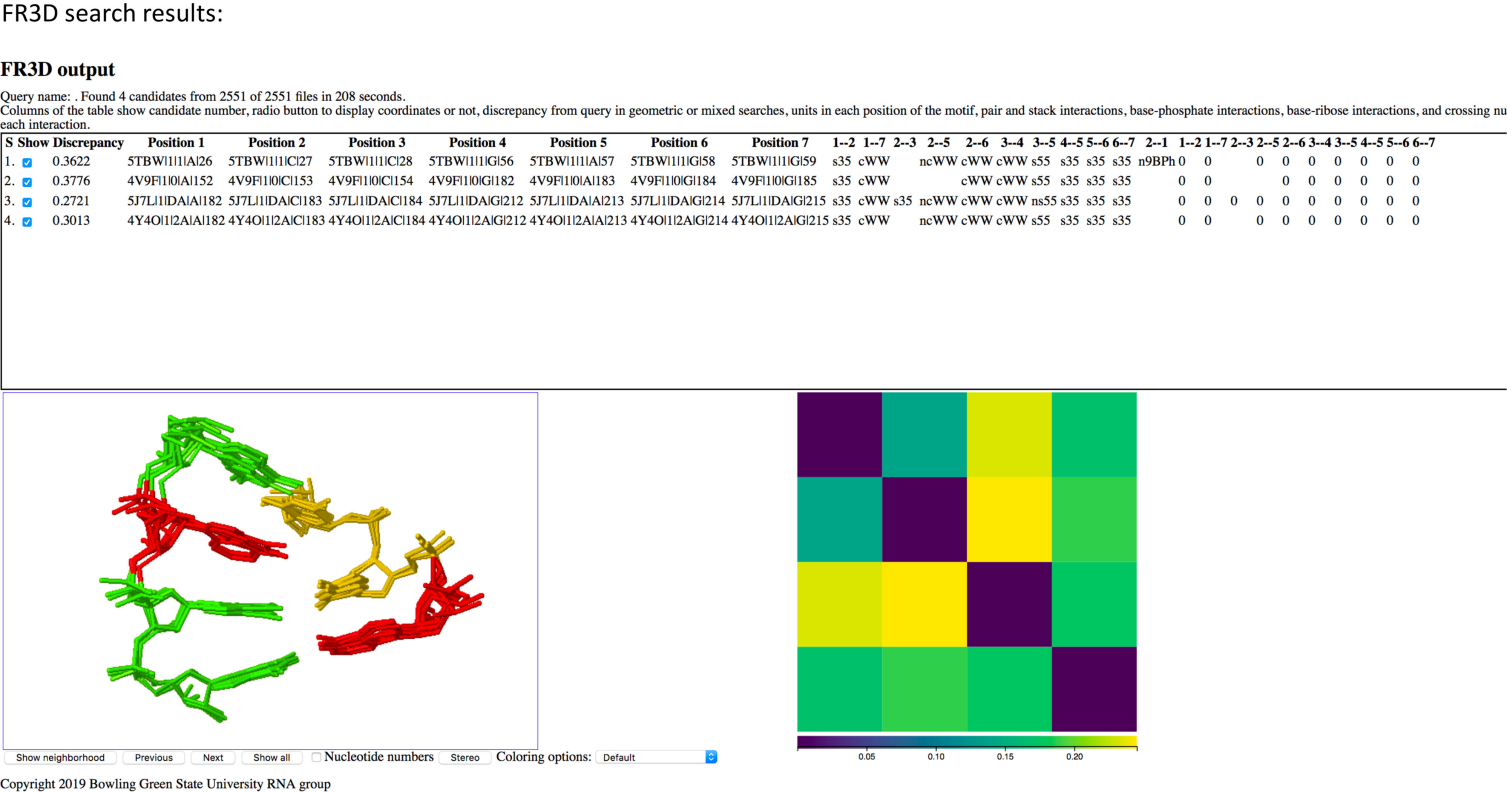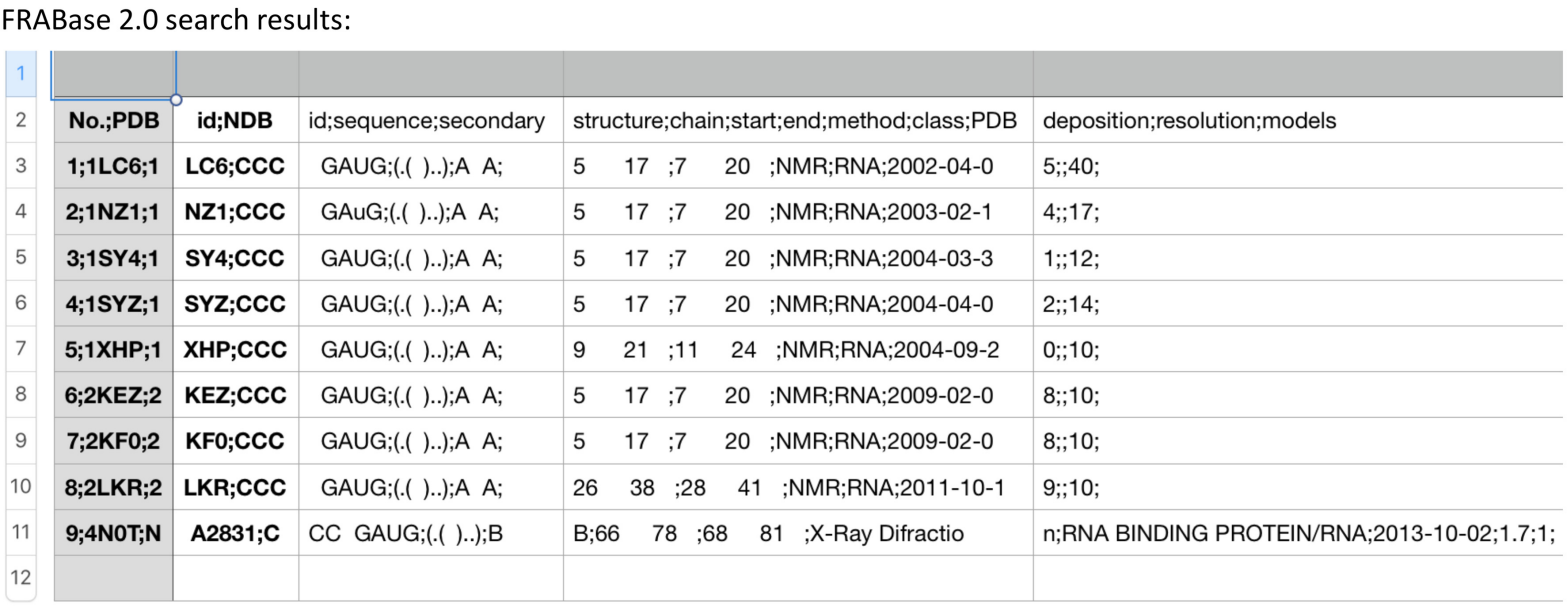# Thermodynamic examination of pH and magnesium effect on U6 RNA internal loop

**DOI:** 10.1261/rna.070466.119

**Published:** 2019-12

**Authors:** Allison A. O'Connell, Jared A. Hanson, Darryl C. McCaskill, Ethan T. Moore, Daniel C. Lewis, Neena Grover

**Affiliations:** Department of Chemistry and Biochemistry, Colorado College, Colorado Springs, Colorado 80903, USA

**Keywords:** RNA 1 × 2 loop, single-nucleotide bulge loop, magnesium ion–RNA interactions, RNA thermodynamics, internal loops, spliceosomal RNA

## Abstract

U6 RNA contains a 1 × 2-nt internal loop that folds and unfold during spliceosomal assembly and activation. The 1 × 2 loop consists of a C_67_•A_79_ base pair that forms an additional hydrogen bond upon protonation, C_67_•A^+^_79_, and uracil (U80) that coordinates the catalytically essential magnesium ions. We designed a series of RNA and DNA constructs with a 1 × 2 loop sequence contained in the ISL, and its modifications, to measure the thermodynamic effects of protonation and magnesium binding using UV-visible thermal denaturation experiments. We show that the wild-type RNA construct gains 0.43 kcal/mol in 1 M KCl upon lowering the pH from 7.5 to 5.5; the presence of magnesium ions increases its stability by 2.17 kcal/mol at pH 7.5 over 1 M KCl. Modifications of the helix closing base pairs from C–G to U•G causes a loss in protonation-dependent stability and a decrease in stability in the presence of magnesium ions, especially in the C68U construct. A79G single-nucleotide bulge loop construct showed the largest gain in stability in the presence of magnesium ions. The DNA wild-type construct shows a smaller effect on stability upon lowering the pH and in the presence of magnesium ions, highlighting differences in RNA and DNA structures. A U6 RNA 1 × 2 loop sequence is rare in the databases examined.

## INTRODUCTION

Eukaryotic translation of proteins requires excision of introns from pre-mRNA to form mature messenger RNA. Nuclear pre–messenger RNA excision, termed splicing, is catalyzed by the spliceosome. The spliceosome is a multimegadalton complex that functions as a ribozyme to excise introns and ligate exons in the process of creating mature messenger RNA (Fica et al. 2013). It is comprised of more than 70 structural proteins as well as five catalytic small nuclear RNAs (snRNAs) designated U1, U2, U4, U5, and U6 (Rappsilber et al. 2002; Zhou et al. 2002). The U6 snRNA must undergo a series of structural rearrangements to assemble and activate the spliceosome and catalyze RNA splicing (Fortner et al. 1993). One structural rearrangement is the unfolding and refolding of a highly conserved internal stem–loop (ISL) in the U6 snRNA (Fortner et al. 1993). The unfolded configuration of the U6 ISL is required for RNA–RNA binding between the U6 and U4 snRNPs, which is necessary for spliceosome assembly (Madhani and Guthrie 1992; Fortner et al. 1993; Johnson and Abelson 2001; Will and Lührmann 2011). The refolding of the U6 ISL allows the U6 snRNP to release the U4 snRNA and bind to the U2 snRNA, a step required for spliceosome activation. The U2/U6 complex then catalyzes splicing via two transesterification reactions (Datta and Weiner 1991; Madhani and Guthrie 1992; Valadkhan and Manley 2003).

**FIGURE 1.**
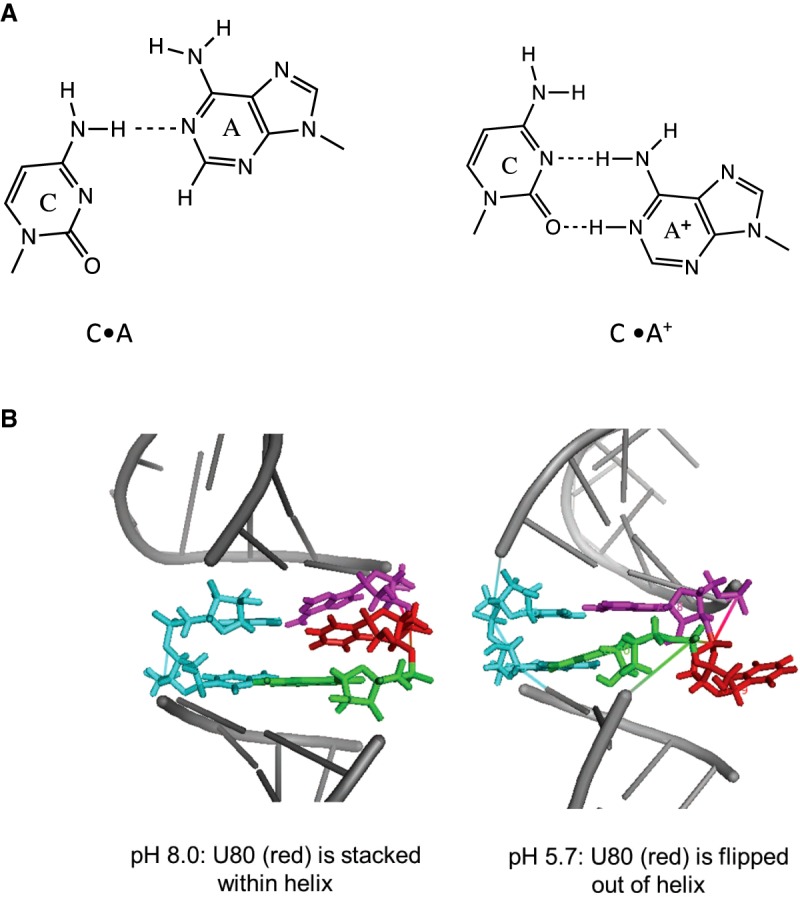
(*A*) The C•A and C•A^+^ base pairs. (*B*) NMR structures of RNA wild-type construct at pH 8.0 (*left*) and pH 5.7 (*right*). Image generated using PyMOL, pH 5.7:pdb 1SYZ, pH 8.0: pdb 2KEZ (Reiter et al. 2004; Venditti et al. 2009).

The U6 snRNA has been extensively studied using NMR and genetic studies (Datta and Weiner 1991; Reiter et al. 2003; Sashital et al. 2003; Blad et al. 2005; Didychuk et al. 2018). The U6 ISL contains a 1 × 2 internal loop that is essential for spliceosome assembly and catalysis (Epstein et al. 1980; Huppler et al. 2002; McManus et al. 2007). The 1 × 2 loop consists of a cysteine•adenosine base pair (*cis* Watson–Crick/Watson–Crick, cWW), with one-hydrogen bond between residues C_67_•A_79,_ and an adjacent unpaired uracil, U80 (numbering according to yeast *Saccharomyces cerevisiae*) (Fortner et al. 1993; Staley and Guthrie 1998; Huppler et al. 2002). This 1 × 2 loop is influenced by both protonation and cation binding (Yean et al. 2000; Reiter et al. 2004; Venditti et al. 2009). The internal loop adenine (A79) has a pK_a_ of 6.5; upon protonation, the C_67_•A^+^_79_ wobble pair will form an additional hydrogen bond (Fig. 1A; Huppler et al. 2002). The NMR structural data shows that A79 protonation induces a conformational change in RNA (Reiter et al. 2004; Venditti et al. 2009). At a pH < 6.0, adenine protonation favors U80 in a “flipped out” conformation, allowing the ISL to assume a near helical confirmation (Fig. 1B; Reiter et al. 2004). NMR performed at a pH of 8 shows U80 is stacked within the helix, disrupting the C•A^+^ pair and forming a new C_67_•U_80_ pair (Fig. 1B; Venditti et al. 2009). Anokhina et al. (2013) used chemical techniques to probe purified human spliceosome RNA structure and found no variability of DMS activity at position A79 under examined conditions, indicating no changes in accessibility at this site.

The U80 in the 1 × 2 internal loop coordinates magnesium ions essential for catalyzing transesterification reactions in splicing RNA (Yean et al. 2000; Fica et al. 2013). NMR studies of the U6 ISL provided initial insights into the structure of this metal-binding domain (Huppler et al. 2002; Blad et al. 2005). Early studies showed sulfur substitution at the U80 pro-S_P_ nonbridging phosphate oxygen abolishes the first step of splicing but can be rescued by thiophilic ions such as cadmium (Yean et al. 2000). Subsequent studies have revealed that U80 pro-S_p_ and pro-R_p_ nonbridging phosphate oxygens coordinate two metal ions and that the U80 pro-S_p_ oxygen is required for both transesterifications, including branching and exon ligation (Fica et al. 2013). Furthermore, stereospecificity of metal binding at U80 is lost at a low pH (Huppler et al. 2002). This suggests that protonation of the adjacent C_67_•A^+^_79_ pair prevents magnesium binding. This study also demonstrated the pK_a_ of A79 decreases by half a pH unit when MgCl_2_ was present and suggested that metal ion binding and protonation were mutually antagonistic (Huppler et al. 2002). Magnesium binding to U6 has been inferred through NMR and thiophosphate modification experiments. Modes of magnesium binding to RNA (inner or outer sphere) are not known and neither are all the ligands that it may interact with in its different structural forms.

In this article, we measured the thermodynamic properties of the wild-type and modified U6 1 × 2 loop derived from *S. cerevisiae* to understand the relationships between sequence, protonation-dependent hydrogen bonding, and magnesium-dependent changes in RNA stability. The wild-type U6 construct was modified at positions C66, C68, and A79 based on yeast genetic studies that showed the importance of these residues in spliceosomal function (McManus et al. 2007). A few DNA 1 × 2 constructs were also designed to better understand the differences between DNA and RNA structures with regards to adenosine protonation and magnesium-dependent stabilization of small internal loops in nucleic acids.

Our experiments show that an additional hydrogen bond in the C_67_•A^+^_79_ in the U6 ISL adds 0.43 kcal/mol to RNA stability in 1 M KCl at pH 5.5. In the presence of 10 mM magnesium ions, all RNA internal loop and bulge loop constructs are additionally stabilized by 0.67–3.89 kcal/mol over 1 M KCl. The wild-type U6 1 × 2 construct shows 2.17 kcal/mol increase in stability in 10 mM magnesium ions over 1 M KCl containing buffer at pH 7. DNA constructs show a small protonation-dependent change in stability and a small magnesium ion stabilization effect.

## RESULTS

### RNA wild-type construct is additionally stabilized at pH 5.5

**FIGURE 2.**
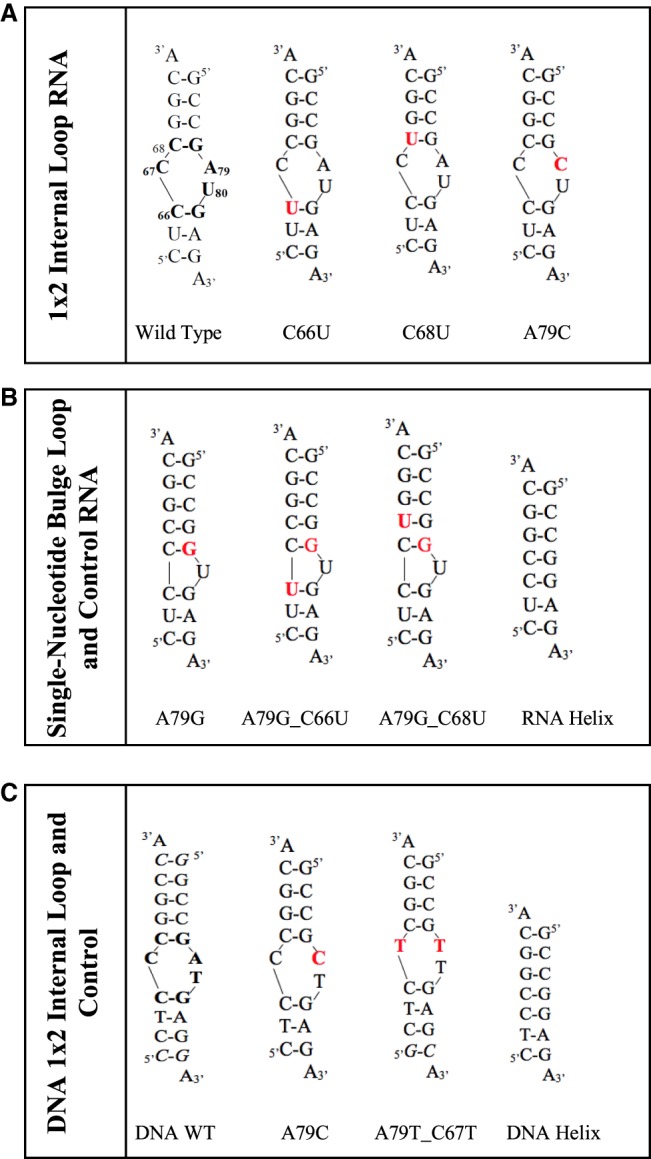
RNA and DNA constructs used in this study. (*A*) The RNA constructs that contain a 1 × 2 nt loop. (*B*) The RNA constructs that contain a single-nucleotide bulge loop and the control helix. (*C*) DNA constructs that contain 1 × 2 nt loop and the control helix. The additional base pairs added to the DNA constructs to increase RNA stability are shown in italics. Motif being examined is shown in bold font on the wild type; modifications to the motifs are in red.

Thermodynamic stability (ΔG^o^_37_) of RNA and DNA constructs (Fig. 2) was measured in 1 M KCl–containing buffer at pH 7.5 or 5.5. The RNA wild-type construct had a Δ*G*^o^_37_ of −9.37 and −9.80 kcal/mol at pH 7.5 and 5.5, respectively (Table 1). An additional 0.43 kcal/mol stability is gained upon lowering the pH, likely because of A79 protonation and formation of an additional hydrogen bond between C_67_•A^+^_79_ (Table 2; Fig. 1A).

**TABLE 1.**
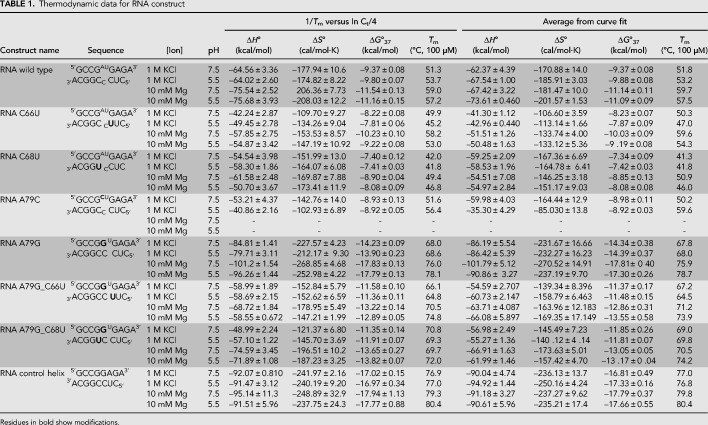
Thermodynamic data for RNA construct

**TABLE 2.**
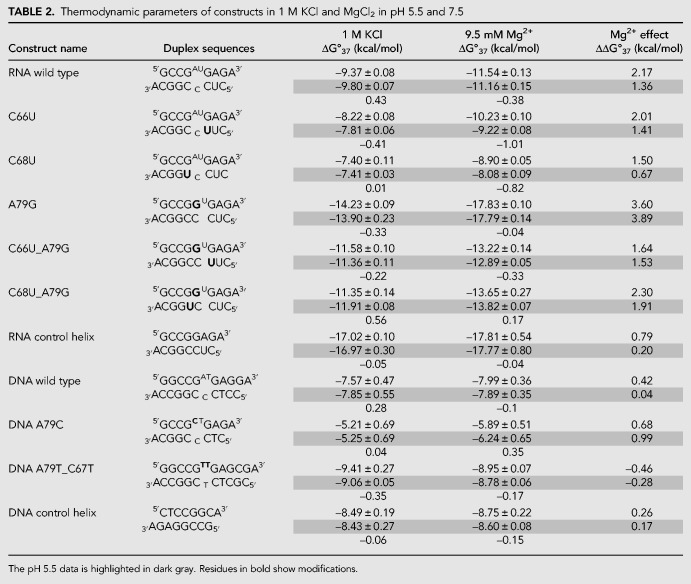
Thermodynamic parameters of constructs in 1 M KCl and MgCl_2_ in pH 5.5 and 7.5

Changing the closing base pairs on the stems from C–G to U•G base pairs, in C66U (5′ helical stem) and C68U (3′ helical stem) constructs, decreases the stability of RNA by 1.15 and 1.97 kcal/mol relative to the wild-type construct in pH 7.5. Neither C66U or C68U show an increase in stability in 1 M KCl in pH 5.5 over pH 7.5 (i.e., no additional protonation-dependent hydrogen bonding as expected for C_67_•A_79_ in wild type), with C66U showing 1.99 kcal/mol and C68U showing 2.39 kcal/mol lowered stability than wild-type construct in pH 5.5. This indicates that the closing U•G base pairs are changing the structural dynamics of the internal loop.

**FIGURE 3.**
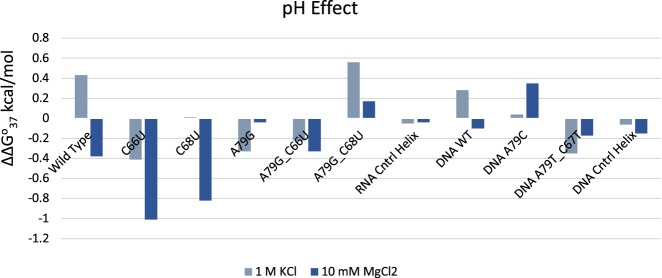
Effect of pH on construct stability. The ΔΔ*G*° values (in kcal/mol) are the difference between Δ*G*°_37_ at pH 5.5 and pH 7.5. Positive values indicate gain in stability at pH 5.5 over pH 7.5.

Changing A79 position to a C (A79C), creates an all-pyrimidine 1 × 2 internal loop with stability that is 0.44 kcal/mol lower than the wild type at pH 7.5, indicating that the wild-type construct has at least a single hydrogen bond between C_67_•A_79_ that is lost upon A79C modification. The magnitude of energy gained and lost upon hydrogen bond formation in the wild-type construct and the A79C construct is identical, albeit for different hydrogen bonding pairs at positions 67 and 79. The A79C construct shows no additional gain in stability in pH 5.5, indicating no C_67_•C_79_^+^ base pair formation (Table 2; Fig. 3).

Changing the A79 to G (A79G) replaces the C_67_•A_79_ with a canonical C_67_–G_79_ base pair and converts the construct to a single-nucleotide (U80) bulge loop; this construct shows a 4.83 kcal/mol gain in stability over the wild-type construct at pH 7.5. Altering the 3′ and 5′ neighbor of the newly formed G–C base pair from a C–G to a U•G wobble pair (A79G_C66U and A79G_C68U) decreases the stability of the RNA by 2.6 and 2.9 kcal/mol, respectively; these constructs still have greater stability than the wild-type construct.

The RNA helix was designed by eliminating the internal loop and has a stability of −17.02 kcal/mol at pH 7.5; the change in pH did not affect its stability (Table 2; Fig. 3).

The DNA wild-type construct (with two additional G–C base pairs in the stem compared to the RNA wild construct) had Δ*G*^o^_37_ of −7.57 kcal/mol, with a gain of 0.28 kcal/mol in stability upon lowering the pH to 5.5 (Table 3). Subsequent modifications to the DNA construct were used to generate all-pyrimidine 1 × 2 internal loops. The formation of a T•T wobble pair (A79T_C67T) increased the stability of the construct by 1.84 kcal/mol at pH 7.5. At pH 5.5, this construct (A79T_C67T) decreased in stability by 0.35 kcal/mol. The A79C construct also does not gain additional stability upon lowering the pH indicating no C•C^+^ formation in the DNA construct as well (Fig. 3). The DNA control helix has a stability of −8.49 kcal/mol and does not gain additional stability upon lowering the pH.

**TABLE 3.**
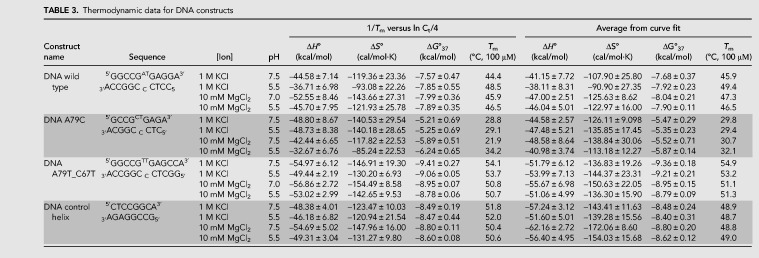
Thermodynamic data for DNA constructs

### All RNA constructs are stabilized in magnesium chloride

**FIGURE 4.**
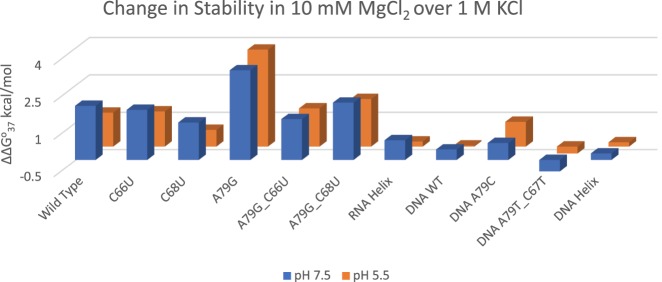
Effects of 10 mM magnesium chloride on construct stability. The ΔΔ*G*° values (in kcal/mol) are shown for two different pH conditions and are calculated as a difference between Δ*G*°_37_ in 1 M KCl and 10 mM MgCl_2_. Positive values indicate a gain in stability in 10 mM MgCl_2_ over 1 M KCl buffers.

All RNA internal loop and single-nucleotide bulge loop constructs examined here were more stable in the presence of magnesium ions versus in 1 M KCl–containing buffers. In 10 mM MgCl_2_ buffer, the RNA wild-type construct gained an additional 1.36 and 2.17 kcal/mol in stability over 1 M KCl in pH 5.5 and 7.5, respectively (Table 2; Fig. 4). The stabilizing effect of magnesium ions ranged from 0.67 to 3.89 kcal/mol for the various RNA constructs, indicating some sequence and structure-specific interactions between magnesium ions and RNA.

### DNA constructs show smaller additional stabilization in magnesium chloride

The DNA wild-type construct gained 0.42 kcal/mol in the presence of magnesium ions at pH 7 and with no additional gain in the lower pH. A79C gained 0.68 and 0.99 kcal/mol in stability in the presence of magnesium ions at pH 7.5 and 5.5, respectively. A79T_C67T had lower stability in magnesium ions as compared to 1 M KCl, indicating that sequence and structure-specific interactions also occur in DNA internal loops.

### Penalties for breaking helix with a single-nucleotide bulge and 1 × 2 internal loop

Disrupting a helical structure destabilizes the RNA. The changes to RNA stability upon the incorporation of internal loops are represented as ΔG^o^_37,loop_ parameters. The ΔG^o^_37, loop_ parameters for various constructs are shown in Table 4. Breaking the RNA helix by a single-nucleotide bulge loop destabilized the RNA more than 1 × 2 internal loop. Additional interactions that are occurring within the 1 × 2 internal loop are compensating for the penalty of breaking the helical structure. The RNA wild-type construct had a ΔG^o^_37,loop_ of 4.39 kcal/mol at pH 7.5 and 3.91 kcal/mol at pH 5.5 where an additional hydrogen bond forms between C_67_•A^+^_79_. At a pH of 7.5, the penalty for 1 × 2 loops ranged from 3.70 to 5.25 kcal/mol. The penalty for breaking the helix with a single-nucleotide bulge loop is in the range of 5.44 to 6.09 kcal/mol at pH 7.5, depending on the composition of the stem.

**TABLE 4.**
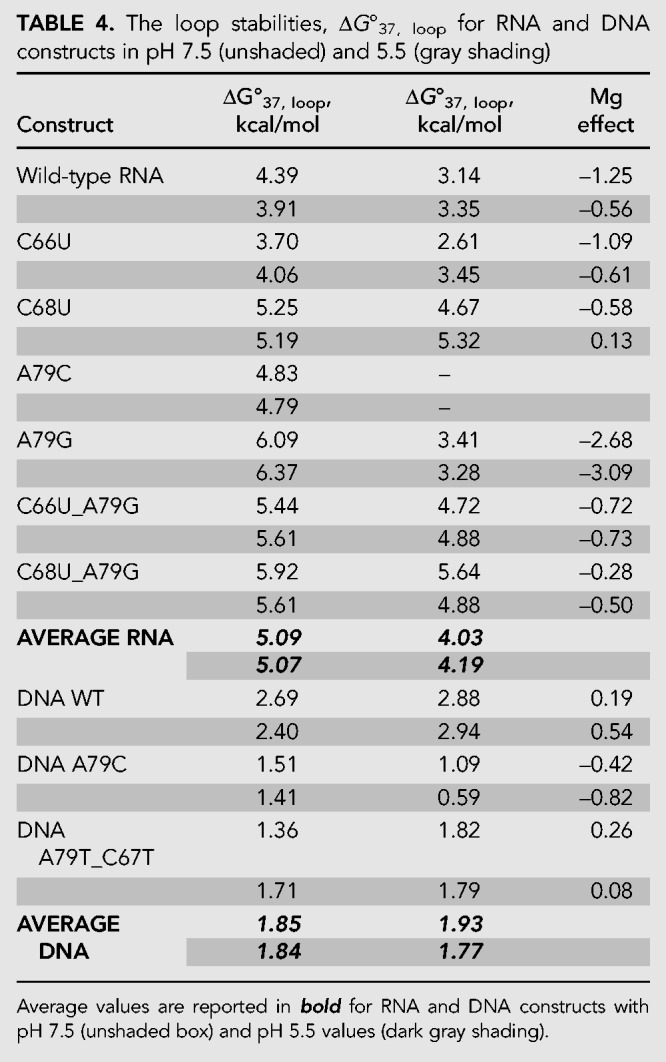
The loop stabilities, Δ*G*°_37, loop_ for RNA and DNA constructs in pH 7.5 (unshaded) and 5.5 (gray shading)

The penalty for disrupting a helical backbone with a loop is smaller in DNA than for RNA. For DNA constructs, a 1 × 2 loop destabilized the helix between 1.36 and 2.69 kcal/mol.

The DNA wild-type construct had the largest penalty for breaking the helical backbone with a Δ*G*°_37,loop_ of 2.69 kcal/mol with a range of 1.36–2.69 kcal/mol at pH 7 for the constructs examined here. Our results show that additional structures are likely forming in A79C and A79T_C67T DNA constructs that contribute to a decrease in the penalty of breaking the helical structure.

### Penalty for breaking the helix is significantly reduced by the presence of magnesium chloride

For all RNA constructs except C68U, the penalty for breaking the helix is lowered in the presence of Mg^2+^, with the Δ*G*°_37,loop_ ranging from 2.61 to 5.64 kcal/mol. The RNA wild-type construct showed a gain in stability (i.e., a decrease in penalty for breaking the helix) of −1.25 kcal/mol (pH 7.5) and −0.56 kcal/mol (pH 5.5) over 1 M KCl (Table 4), indicating that the interactions with magnesium ions are significant and may compensate for some loss in stability depending on the sequence and structures formed by the internal loops. The A79G construct shows the greatest impact of magnesium interactions on the penalty. In this single-nucleotide bulge loop construct, interactions with magnesium show −2.68 kcal/mol (pH 7.5) and −3.09 kcal/mol (pH 5.5) gain in stability in the presence of magnesium ions over 1 M KCl.

For DNA constructs, the effects of Mg^2+^ on the Δ*G*^o^_37, loop_ parameters ranged from 0.59 to 2.94 kcal/mol. The DNA A79C construct was stabilized in the presence of magnesium (Table 4).

### Structural database analysis

WebFR3D and FRABase, were utilized to search for the U6 1 × 2 RNA motif in the structural databases (Popenda et al. 2010; Petrov et al. 2011). Using the FR3D program, a 7-nt sequence, the 1 × 2 internal loop and two closing C–G base pairs, yielded four matches from among 2551 structures searched in the databases. All four sequences were from the large subunit of the ribosomal RNA: 70 S ribosomal RNA from *Escherichia coli*, *Thermus theromphilus*, and *Haloarcula morismortui* and one from 80S ribosome from yeast 80S. All contained a canonical closing base pair on the helical stems, with three containing it near a Watson–Crick base pair A•C (ncWW) (see Appendix 1). When U•G closing pairs were searched at either end of the helix, no similar structures were identified. Using FRABase, when the same 7-nt search was performed, all nine structures identified were from U6 RNA structures (see Appendix 1). No structures were identified containing U•G closing base pairs. A generic search for a 1 × 2 internal loop shows that it is found 2896 times in the structural databases using the FRABase search. These database search results underscore that U6 ISL is a relatively rare structural motif.

## DISCUSSION

### Hydrogen bonding, protonation, and closing base pairs

Thermodynamic analysis of the wild-type RNA construct showed that the C_67_•A_79_ base pair likely has a single hydrogen bond at pH 7 with energetics of 0.45 kcal/mol. Protonation of A79 allows for another hydrogen bond formation that increases RNA stability by 0.43 kcal/mol. Previous studies show that adenine protonation contributes 0.7 ± 0.3 kcal/mol to the overall stability of the construct in this RNA and is within the range measured here (Reiter et al. 2003). The strengths of individual hydrogen bonds have also been measured previously and are shown to be context-dependent, with the strength of hydrogen bond in the hairpin loop being <0.7 kcal/mol (Turner et al. 1987; SantaLucia et al. 1992).

Upon modification of adenine 79 to a cytosine (A79C), the pH-dependent gain in stability is lost, indicating that there is no C^+^•C base pair formation in the sequence and solution context examined here. Additional hydrogen bonding has been shown to stabilize the 1 × 2 internal loops previously, especially those containing A•G and U•U noncanonical base pairs (Schroeder et al. 1996).

In RNA constructs that contain C–G to U•G modifications (C66U and C68U), we examined the role of C–G closing base pairs in the formation of the C_67_•A^+^_79_ base pair. The C66U and C68U constructs did not gain stability upon lowering the pH, implying that the modifications to both the 5′ and 3′ base pairs flanking the noncanonical A•C base pair have an impact on the RNA structure of the loop and formation of the hydrogen bonds between C_67_•A_79_. A significant decrease in stability is seen for U•G substitutions at positions 66 and 68, with a greater impact on RNA stability seen for the C68U construct. It is possible that the closing U•G base pair alters the loop structural dynamics in the C68U construct. In single-nucleotide bulge loops, closing G•U base pairs destabilized the RNA by 0.5–4 kcal/mol and showed non–nearest neighbor effects (Blose et al. 2007).

### Magnesium binding

The 1 × 2 loop is expected to create a Mg^2+^ binding pocket in the U6 ISL. Upon protonation of A79, U80 gets flipped into the major groove allowing the RNA to become close to an A-form helix, causing a loss of the binding pocket. Hence, Mg^2+^ binding was suggested to be lost upon A79 protonation (Huppler et al. 2002; Reiter et al. 2004). The pK_a_ of A79 is expected to decrease in the presence of magnesium ions (Huppler et al., 2002), suggesting that magnesium prevents protonation and that both protonation and magnesium binding are mutually antagonistic. In the presence of Mg^2+^, the RNA wild-type construct is more stable at pH 7.5 over pH 5.5 by 0.43 kcal/mol. Thus, the gain in stability by lowering the pH is no longer seen when the RNA wild-type construct is in Mg^2+^. This suggests that at 10 mM magnesium concentrations, the binding of magnesium prevents A79 protonation, and therefore we do not detect the increase in stability we expect when the pH is lowered. However, the A79G construct that mimics the protonated form of the ISL and is likely to form the “flipped out” U80 structure showed the greatest gain in stability upon addition of magnesium. It is possible that the unstacked conformation of U80 positions the phosphates for even more favorable interactions with magnesium ions. The binding of magnesium provides significantly greater stability for the construct mimicking the “flipped out” conformation. This might indicate that the conformations that are present when A^+^•C is forming are important in sites and/or selectivity of magnesium–RNA interactions. Our data suggest that C68 may also aid in magnesium binding. Although both the RNA wild-type and C66U construct display similar gains in stability in the presence of magnesium ions (ΔΔ*G*^o^_37_, Table 3), stability of the C68U construct in magnesium ions is 0.63 kcal/mol lower in both pHs—either a direct measure of interactions or an indirect effect of changes in RNA conformations that bind to magnesium ions. Binding of magnesium ions to RNA containing small loop motifs provides an additional 1–2 kcal/mol stability over 1 M salt (Serra et al. 2002; Carter-O'Connell et al. 2008; Furniss and Grover 2011; Strom et al. 2015).

### A79G_C68U: thermodynamics versus genetics

The A79G mutation in U6 snRNA in *S. cerevisiae* causes cold sensitivity, presumably because of hyperstabilization of the ISL preventing U4/U6 dimerization (Fortner et al. 1993). McManus et al. reported that C68U mutations rescued the A79G mutant yeast, whereas C66U mutations were lethal when in combination with A79G. We set out to determine if this difference in phenotype could be explained by alterations in the ISL stability. In studying these modifications alone, both modifications reduced stability compared to the RNA wild-type construct. The C66U construct was more stable than the C68U construct. Even though changes in RNA stability upon C–G to U•G modifications were greater when present in combination with the A79G construct, both constructs containing dual modifications (C66U_A79G and C68U_A79G) had stabilities higher than the stability of the RNA wild-type construct. This suggests that the effects of the A79G, the C66U, or the C68U mutations seen in yeast are not thermodynamic in nature but likely due to changes in RNA dynamics needed for other intermolecular interactions, such as binding with the Prp24 protein that chaperones U4/U6 binding during spliceosome assembly (Martin-Tumasz and Butcher 2009; Martin-Tumasz et al. 2011; Montemayor et al. 2014, 2017; Didychuk et al. 2018) or binding to PRP45, the yeast homolog to SKIP protein, which coordinates and stabilizes the final stages of spliceosome complex maturation (Figueroa and Hayman 2004; Haselbach et al. 2018). It is also possible that tighter magnesium binding stabilizes particular RNA or magnesium conformations that prevent proper folding and unfolding of RNA or availability of magnesium ions for catalytic reactions of the spliceosome.

### U6 1 × 2 internal loop is a rare structural motif

WebFR3D and FRABase searches for an RNA motif that contains similar structure and sequences to U6 RNA show that the 1 × 2 internal loop motif of U6 is rare (Popenda et al. 2010; Petrov et al. 2011). This is not surprising given the complex structural dynamics that U6 ISL undergoes in performing its various spliceosomal functions (Didychuk et al. 2018).

### Internal loops in RNA

The penalty for breaking the RNA helix with a 1 × 2 internal loop is dependent on the sequence of the internal loop, the pH, and the presence of magnesium ions. The penalty for breaking the RNA helix with the wild-type loop sequence was greater at pH 7.5 than at pH 5.5. This can be attributed to protonation dependent stabilization of the helix that leads to a single-nucleotide-like bulge loop. The literature value for the 1 × 2 internal loop penalty in 1 M NaCl for Δ*G*^o^_37,loop_ is 2.2 ± 0.1 kcal/mol (Schroeder et al. 1996; Phan et al. 2017). The individual Δ*G*^o^_37,loop_ measured values in Badhwar et al. varied between 2.47 and 5.29 kcal/mol for the 1 × 2 bulge loop identical to the U6 wild-type construct when placed in different helical stems (Badhwar et al. 2007), indicating that non–nearest neighbor effects play a significant role in RNA stability. The Δ*G*^o^_37,loop_ measured here in 1 M KCl had a range of 3.70–5.25 kcal/mol in pH 7.5 and 2.61–5.32 kcal/mol for pH 5.5 in the same helical context. Thus, in the context of U6 RNA, the 1 × 2 bulge is significantly destabilizing in 1 M KCl. In the presence of magnesium ions, the penalty for breaking the helix decreases by as much as 1.25 kcal/mol over 1 M KCl. The C68U construct was uniquely destabilizing (Δ*G*^o^_37,loop_ = 5.25 kcal/mol), likely indicating that the G•U base pair at the end of the helix causes significant changes to the loop, perhaps leading to a potential 2 × 3 internal loop, for which Δ*G*^o^_37,loop_ of 3.5 kcal/mol is utilized in prediction models (Phan et al. 2017). The presence of magnesium ions or lowering the pH did not decrease the penalty. Berger et al. report that 2 × 2 internal loops closed by G•U pairs were significantly less stable than predicted (Berger et al. 2018). Greater penalties are seen when a helical backbone is disrupted by an unpaired uracil with additional G•U modifications in the stems. Replacing the terminal C–G base pair with a U•G base pair (C66U_A79G) resulted in a 5.44 kcal/mol penalty. Replacing an adjacent internal base pair (C68U_A79G) resulted in a 5.92 kcal/mol penalty. This difference implies that stem G•U modifications are more destabilizing than is expansion of the internal loop. Alternatively, Schroeder et al. suggested that U•U base pairs stabilize 2 × 3 loops. The C66U_A79G construct could form a U•U base pair, and, as such, this interaction could provide additional stability to the construct in comparison to the C68U_A79G construct (Schroeder and Turner, 2000). We did not conduct structural studies to test structures formed in the internal loops.

Previous reports on small internal loops in DNA show that the free energy contribution of the loop depends on the nucleotide sequence, with a penalty of 3.2 kcal/mol for internal loop of 3 nt being utilized in the nearest neighbor models (SantaLucia and Hicks, 2004; Tran and Cannon, 2017). Noncanonical T•T base pair formations have been shown to reduce the penalty for DNA loop motifs (Tran and Cannon, 2017).

In this study, the penalty for breaking the RNA helix with the single-nucleotide pyrimidine bulge loop is on average 5.8 kcal/mol and is ∼1.4 kcal/mol lower in the presence of magnesium ions, indicating specific interactions with magnesium ions for certain sequences. Literature values report a penalty of 3.4 ± 0.7 kcal/mol for single-nucleotide bulge loops (Znosko et al. 2002; Strom et al. 2015). The current prediction models use a penalty of 3.8 kcal/mol for all single-nucleotide bulge loops (Mathews et al. 1999).

### Differences in RNA and DNA constructs

Our results show that the differences in RNA and DNA constructs are significant even in small nucleic acid constructs. The differences in free energy are expected because of increased hydrogen bonding due to 2′-hydroxyl group in RNA, differences in stacking energies due to differences in A- and B-form helices, etc. We have specifically been interested in quantifying the differences in ion interactions, particularly magnesium-based stabilization of RNA structures. Whether magnesium interaction in RNA is due to the closer phosphate–phosphate distances (5.9 Å in RNA vs. 7 Å in DNA) or due to site-specific binding, various studies in our laboratory show a trend that RNA containing bulge loops and internal loops is additionally stabilized in the presence of magnesium ions (Carter-O'Connell et al. 2008; Strom et al. 2015). Experiments performed on duplexed RNA–RNA, DNA–RNA, and DNA–DNA also show changes in stability in different ions and ionic strengths (Nakano et al. 1999; Gu et al. 2013).

## MATERIALS AND METHODS

### Construct design

U6 snRNA constructs were derived from *S. cervisiae* U6 ISL (Huppler et al., 2002). Two noncomplementary strands were designed for ease of modifications. All constructs were designed to yield one thermodynamically favored structure in solution using the nearest neighbor model as utilized by the RNAStructure prediction program (Mathews et al. 1999). The presence of an ^3′^A overhang additionally stabilizes the RNA constructs and prevents the end base pairs from fraying (Freier et al. 1985).

Biologically relevant modifications in U6 snRNA constructs were designed based on genetic studies examining U6 ISL point mutations on yeast phenotypes (McManus et al. 2007). An A79G mutation changes the C•A wobble pair to a canonical G–C base pair, which results in a cold-sensitive yeast strain (Fortner et al. 1993). This cold-sensitive phenotype can be exacerbated or alleviated by modifications that are expected to increase or decrease the internal loop's stability, respectively. A C68U modification alleviates this cold-sensitive phenotype. This modification converts the base pair 3′ to the loop from a C–G to a U•G wobble pair. A C66U modification, which converts the C–G to a U–G wobble pair 5′ to the loop, exacerbates the A79G-induced cold-sensitive phenotype (McManus et al. 2007). Based on these observations, we designed RNA constructs that represent the wild type sequence of the U6 ISL as well as constructs containing the A79G, C66U, and C68U modifications alone or in combination to determine the effects these modifications have on stability, protonation, and magnesium binding. An RNA construct was designed with an A79C modification to elucidate the effects of alternate base pairs within the U6 ISL loop. An RNA helical construct that lacked the loop nucleotides was also designed. Thus, four constructs with a 1 × 2 internal loop (Fig. 2A) and three constructs with a single-nucleotide bulge and a control helix (Fig. 2B) were examined using thermal denaturation experiments.

Three different DNA constructs were also designed to understand the 1 × 2 internal loops in DNA (Fig. 2C). The DNA wild-type construct contains the DNA equivalent of the 1 × 2 internal loop present in the U6 ISL. Two additional constructs contain A79C and combination A79T/C67T modifications. Two of the three DNA 1 × 2 internal loop constructs have additional G–C base pairs on either stem to increase the construct stability; these additional base pairs are shown in italics.

### Oligomer purification

RNA oligomers were purchased from Dharmacon, Inc. DNA oligomers were purchased from DNA Technologies, Inc. RNA and DNA was purified using thin-layer chromatography (TLC). For TLC purification, a Baker Si500 silica plate and running buffer of 6:3:1 (v/v/v) 1-propanol, 30% ammonium hydroxide, and water was used. Oligomers were extracted from silica with water and then spin filtered to remove excess silica. Samples were dried by lyophilization using SPD1010 SpeedVac System (Savant). Samples were deprotected with 100 mM acetic acid adjusted to pH 3.8 with TEMED (Dharmacon, Inc). Samples were desalted with C18 Sep-Pak column (Waters WAT020515) using 5 mM sodium bicarbonate loading buffer (pH 6). Elution was performed twice with 2 mL of 30% acetonitrile and once with 100% acetonitrile. Oligomers were collected, dried, and suspended in a designated buffer. Concentrations were calculated using extinction coefficient derived from pairwise values for nearest neighbors.

### Thermal melting experiments and data analysis

Thermodynamic data was obtained using a Cary 100 Bio UV Visible Spectrophotometer fitted with 6 × 6 Peltier unit. Samples were prepared with equimolar amounts of each RNA or DNA strand. Buffers consisted of 10 mM cacodylic acid, 0.5 mM EDTA, and 10 mM KCl with either 1 M KCl or 10 mM MgCl_2_ at pH of 7.5, 7, or 5.5. The concentrations of divalent ions were established by complexometric titrations or atomic absorption spectroscopy (with the detection limit in low- to the submicromolar range, respectively). Thermodynamic analyses performed here are similar to those in previous work and allow for determining changes in RNA stabilities with respect to its own stability under different ionic conditions, allowing for a comparative analysis between different RNA constructs (Carter-O'Connell et al. 2008).

Thermodynamic data were collected as previously described (Carter-O'Connell et al. 2008). The constructs were melted as a duplex in 1:1 concentration ratio using an average extinction coefficient at the rate of 1°C/min between 0°C and 100°C. A 30- to >50-fold range of concentrations was tested in nine individual thermal denaturation experiments by varying cuvette path length between 0.1 and 1 cm. RNA and DNA absorbance was measured at a wavelength of 260 nm. The melt curves generated by thermal denaturation experiments were analyzed using the two-state model (Xia et al. 1998; Mathews et al. 1999). Linear sloping baselines and temperature-independent enthalpy and entropy values were assumed to generate thermodynamic parameters using the Meltwin program to fit individual melting curves (McDowell and Turner 1996). Temperature independence of enthalpy in the presence of magnesium ions has not been established. Thermodynamic parameters generated by individual curve fit were compared with those generated by van't Hoff analysis [*T*^−1^ vs. log (C_T_/4)] plot for non-self-complementary RNA using the following equations (where *T*_m_ is the melting temperature, *C*_T_ is the total concentration of RNA, and R is the gas constant). Thermodynamic data reported is from the van't Hoff analysis, with the exception of the C68U_A79G construct where curve fit data was used.

$$T_{m}^{-1}=2.303\;\left(\frac{R}{\Delta H^{\circ }}\right)\;\ln\left(\frac{C_{T}}{4}\right)+\;\left(\frac{\Delta S^{\circ }}{\Delta H^{\circ }}\right),$$

$$\Delta G^{\circ }=\;\Delta H^{\circ }-T\Delta S^{\circ }.$$

### Internal and bulge loop penalty calculations

Disrupting a helical structure destabilizes the RNA. The changes to RNA stability upon the incorporation of internal loops are represented as Δ*G*^o^_37,loop_ parameters. For a single-nucleotide bulge (Fig. 1B), the assumption is that the helical base stacking is not disrupted (Jaeger et al. 1989; Blose et al. 2007) and, hence, the calculations are performed as

$$\Delta G_{37,\;\mathrm{loop}}^{\circ }=\Delta G_{37,\;\mathrm{RNA}\;\mathrm{with}\;\mathrm{loop}}^{\circ }-\Delta G_{37,\;\mathrm{helix}}^{\circ }.$$

For RNA constructs containing the same RNA backbone as the RNA control helix (wild-type, C66U, and C68U constructs), the measured Δ*G*^o^_37_ of the RNA control helix was used for Δ*G*^o^_37, helix_. Constructs containing the A79G modification have an additional C–G base pair in their helix and are predicted to contribute −3.3 kcal/mol to the helix (Xia et al. 1998; Mathews et al. 1999). For constructs containing modification to the helix (those containing G•U modifications in the stem), the measured Δ*G*^o^_37, helix_ value was used to calculate the helix stability by changing the appropriate nearest neighbor parameters (Chen et al. 2012).

For 1 × 2 internal loops, the calculations for the Δ*G*^o^_37, loop_ parameter were performed with the energetics of disrupted base stacking considered (Jaeger et al. 1989; Xia et al. 1998; Znosko et al. 2002; Chen et al. 2012):

$$\Delta G_{37,\;\mathrm{loop}}^{\circ }=\Delta G_{37,\;\mathrm{RNA}\;\mathrm{with}\;\mathrm{loop}}^{\circ }-(\Delta G_{37,\;\mathrm{helix}}^{\circ }-\Delta G_{37,\;\mathrm{disrupted}\;\mathrm{base}\;\mathrm{stacking}}^{\circ }).$$

The 1 × 2 internal loops also have modifications in the helix, and the calculations for the Δ*G*^o^_37, loop_ parameter were performed with the energetics for new helical parameters that included modifications of appropriate nearest neighbor parameters for G•U base pairs.

The nearest neighbor parameters from Xia et al. (1998) and Chen et al. (2012) were utilized for disrupted base stacking in RNA (Xia et al. 1998; Chen et al. 2012). The calculations for DNA utilized the nearest neighbor parameters from SantaLucia et al. (SantaLucia et al. 1996).

Appendix 1 The 7-nt sequence shown below was searched using FR3D and FRABase 2.0.

        ^5′^C ^C^ C^3′^

        ^3′^G_UA_G^5′^